# Inflammation and hematopoietic stem cells aging

**DOI:** 10.1097/BS9.0000000000000063

**Published:** 2020-11-17

**Authors:** Hanqing He, Jianwei Wang

**Affiliations:** School of Pharmaceutical Sciences, Tsinghua University, Beijing 100084, China

**Keywords:** Inflammation, Hematopoietic stem cells aging

## Abstract

Hematopoietic stem cells (HSCs) replenish all lineages of blood cells throughout the lifespan. During aging, the repopulation capacity of HSCs declined, and aged HSCs display a tendency for myeloid differentiation. Several intrinsic and extrinsic factors have been identified to promote HSCs aging. In this review, we focus on the contribution of aging-associated inflammation in provoking HSCs aging and discuss the future research direction of inflammation and HSC aging.

## 1 INTRODUCTION

**FIGURE 1:**
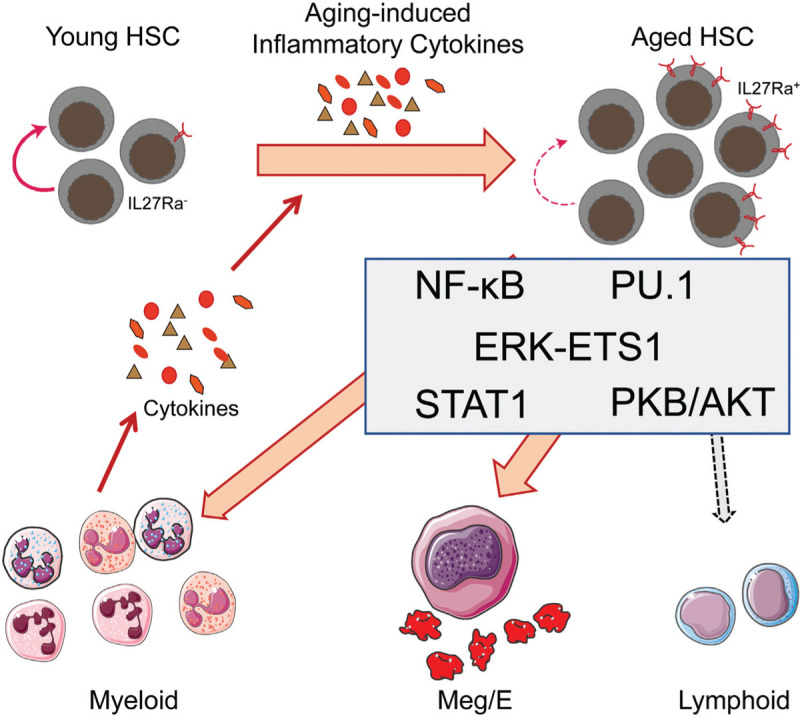
Inflammation promotes hematopoietic stem cells (HSCs) aging. Aging-related inflammatory cytokines such as TNFα, IL-1β, IL-6, IFNα, and IFNγ promote HSCs differentiated into myeloid cells, and newly generated myeloid cells lead to more inflammatory factors production and eventually form a vicious circle.

**FIGURE 2:**
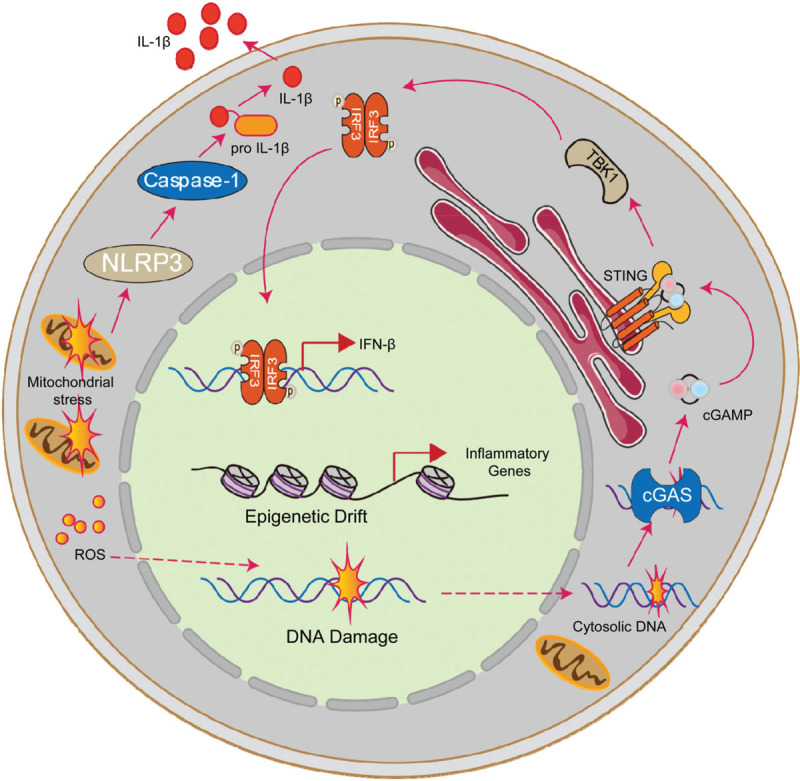
Canonical HSC aging pathways exacerbates inflammatory environment. In the aging context, canonical aging pathways could exacerbate the inflammatory environment. On the one hand, incompletely repaired DNA such as cytosolic DNA fragments activate the cGAS-STING-TBK1-IRF3 pathway, leading to IFNβ production. On the other hand, epigenetic information changes increased the expression of inflammation-related genes. Moreover, mitochondrial stress, in addition to producing reactive oxygen species which attacks DNA and other macromolecules, could also trigger inflammation via the NLRP3-Caspase-1 cascade.

Aging is a biological process which is not modulated by evolutionary selection.^1,2^ During the postnatal development age, the evolutionary selective pressure renders the developmental pathways orchestrated to exhibit the optimal fitness of the organism. However, in the post-reproductive stage, the functional role of the selective pressure on the maintenance of the organism is relatively limited. Therefore, one signaling pathway may perfectly be adapted to the embryonic and postnatal development in the young age of the organism. However, in the post-reproductive age, it is no longer suitable for the organism and even promotes aging. One example is inflammation. In physiological conditions, inflammation facilitates immune-defense and plays a very important role in tissue repair.^3,4^ However, severe inflammatory reactions such as inflammatory cytokine storms caused by viral infections are fatal to organisms.^5^ Besides, elderly people often suffer from chronic inflammation, meanwhile, low-grade chronic inflammation is also considered to be the driving force of aging.^6^ Hematopoietic stem cells located at the top hierarchy of the hematopoietic system, with the capacity of multiple-lineage differentiation and self-renewal, and are responsible for replenishing all blood cells throughout the lifespan.^7^ During aging, the repopulation ability of hematopoietic stem cells declined, and aged HSCs display a tendency for myeloid differentiation.^8–11^ Myeloid cells are considered to be the main source of inflammatory cytokines in the bone marrow and peripheral blood,^12^ and aged HSCs generated more myeloid cells that further exacerbated the inflammatory microenvironment of bone marrow. Recent studies (including ours) have shown that aging-associated chronic inflammation further promotes hematopoietic stem cell aging. In this review, we focus on the following aspects: (1). the molecular mechanism of inflammation in promoting HSC aging; (2) the crosstalk between inflammation and the canonical HSC aging pathways. We also discuss the future research direction of inflammation and HSC aging (Figs. 1 and 2).

## 2 INFLAMMATION PROMOTES HSCS AGING

Accumulating studies reveal that pro-inflammatory cytokines increased in the bone marrow of the elderly and aged mice.^13^ Many studies indicate that inflammatory cytokines are the main driving factors of myeloid-biased differentiation. Pietras et al reported that chronic interleukin-1 (IL-1) stimulation induced HSCs myeloid-biased differentiation, which depends on the nuclear factor kB (NF-κB) signaling and its downstream target PU.1 mediated gene program. Moreover, the continuous exposure of IL-1 declines the self-renewal ability of HSC.^14^ In another study, Ho and his colleagues also found that IL-1a and IL-1b were significantly increased in the bone marrow of aged mice.^15^ Furthermore, they identified that inflammatory factor interleukin-6 (IL-6) increased in the aged bone marrow. Mechanically, they verified that the inflammatory microenvironment of bone marrow promotes megakaryocyte differentiation via β2-AR-IL-6 signaling, which indicates that the IL-6 increment is a reason of megakaryocyte differentiation bias in aged HSCs.^16^ Tumor necrosis factor (TNFα) is a well-established pro-inflammatory cytokine whose stimulation impairs HSCs function. Pronk et al reported that targeting deletion of tumor necrosis factor receptor (TNFR) increased the capacity of HSCs self-renewal.^17^ In another recent study, Yamashita et al demonstrated that the transient exposure of TNF protected HSCs from necroptosis. This pro-survival function depends on NF-κB-p65- cIAP2 cascade downstream of TNFR.^18^ Nevertheless, TNF stimulation drives HSCs differentiated into the myeloid cells, and long-term TNF exposure resulted in the inactivation of NF-kB and render HSCs into necroptosis.^18^

In addition to facilitating myeloid differentiation and impairing self-renewal, inflammatory cytokines can also injure HSCs directly via promoting proliferation and introducing DNA damage. IFNs stimulation renders HSCs escaped from the quiescence, thereby impairing the capacity of HSCs self-renewal. Essers et al reported that HSCs efficiently exit G_0_ phase upon type I interferon stimulation.^19^ HSCs response to interferon-α (IFNα) subsequently enter into cell cycle, which depends on the phosphorylation of STAT1 and PKB/Akt, and IFNα targeted genes expression.^19^ A similar phenomenon also observed in HSCs response to type II interferon and chronic infection.^20^ In another study, Dagmar et al reported that inflammatory stimulus polyinosinic:polycytidylic acid (pI:pC), a mimic of viral infection and activated a type I interferon response, triggers HSCs exit from quiescence, and introduces proliferation-induced DNA damage which ultimately results in HSCs attrition.^21^

In addition to specific inflammatory cytokines, hematopoietic stem and progenitor cells (HSPCs) can sense TLR stimulation such as LPS directly, resulting in accelerating myeloid cell expansion in vitro^22^ and in vivo.^23,24^ Recently, Zhao et al demonstrated that short-term HSCs and multipotent progenitors can produce a large amount of cytokines via activating NF-kB signaling in response to TLR stimuli.^25^ Remarkably, they found that the cytokine production ability of HSPCs were stronger than mature immune cells. Based on the above evidence, we prudently speculate that bone marrow located HSPC itself may also directly respond to inflammatory stimuli and contribute to the aging-related inflammatory microenvironment.

More importantly, several recent studies observed that the response of HSCs to inflammatory stimuli is heterogeneous in young and aged HSCs. Recently, Mann et al reported that when aged HSCs are exposed to LPS, they tend to generate more myeloid cells. This tendency is a cell-intrinsic mechanism that may be regulated in part by Ikzf1, Klf5, and Stat3.^26^ In line with this observation, Chen et al showed that aged HSCs reveal increased NF-κB activity, the activated NF-κB may also contribute to the increased myeloid output of inflammatory exposed aged HSCs.^27^

Serial of inflammatory cytokines upregulated in aged bone marrow, leading to the activation of inflammatory response. However, there is another situation that has been largely ignored: the cytokine concentration remains unchanged, but the expression of cytokine receptor upregulated in HSCs, fueling HSCs to receive more inflammatory signals, which in turn causes HSC aging. A recent study published in BLOOD focused on the change of cytokine receptors in aged HSCs and identified a novel mechanism controlling changes in HSC fate choices during inflammation and aging. They show that aging-related inflammation promotes HSC aging via a TNFα→ERK→ETS1→IL27Ra pathway.^11^

IL-27 is a cytokine composed of EBI3-P28 heterodimer.^28^ Previous studies have shown that artificially overexpressed IL-27 promotes HSC differentiated into myeloid.^29^ In this study, the authors found that the concentration of IL-27 did not change with aging both in peripheral blood and bone marrow, indicating that IL-27 itself is not the driving force of HSC aging. In the aging context, IL-27-IL27Ra signal impairs HSCs may mainly through overexpressing IL27Ra via TNFα-ERK-ETS1 cascade, resulting in that aged HSC receives more IL27 stimulation, which eventually declines HSC function. The authors also found that the expression of inflammation-related genes was more active in IL27Ra^+^ HSCs, which is one of the endogenous mechanisms that deteriorate HSCs function. However, the signal pathway downstream of IL27Ra, especially the intracellular signals activated by IL-27-IL27Ra engagement which directly damaged HSCs under aging conditions, still needed for further research.

Aging-related inflammation promotes HSCs differentiated into myeloid cells, and newly generated myeloid cells lead to more inflammatory factors production and eventually form a vicious circle. What factors triggered this vicious circle? Future research needs further exploration.

## 3 CROSSTALK BETWEEN INFLAMMATION AND THE CANONICAL HSC AGING PATHWAYS

Many intrinsic factors that cause HSC aging can also activate inflammatory pathways. DNA damage and replication stress are the well-known driving force of HSC aging. Previous studies reveal that the accumulation of DNA damage directly deteriorates HSCs function. On the other hand, incompletely repaired DNA such as cytoplasmic DNA fragments can act as inflammatory stimuli to activate the cGAS-STING pathway, leading to IFNβ production and other inflammation cytokine generation.^30^ In addition to cGAS-STING activated type │ interferon production, intracellular dsDNA derived from double-strand break can also activate AIM2 (Absent in melanoma-2) inflammasome and induce Caspase-1 dependent maturation and secretion of IL-1β and IL-18.^31^ Apart from directly detected by sensors like cGAS and AIM2, DNA damage can also induce type-1 interferon production through a cell-autonomous manner which mediated by ATM-IKKα/β-IRF3 cascade and ultimately promotes stem cells senescence.^32^

In addition to DNA damage, reprogramming in epigenetic information is another well-known cause of HSC aging. Chambers et al reported that in the aging context, due to changes in epigenetic information, the expression of inflammation-related genes is increased, which activates inflammation signals ultimately.^33^

Autophagy is a quality controlling mechanism of organelles and macromolecules, which is crucial for maintaining proteostasis and cellular homeostasis. During aging, the activity of autophagy is compromised.^34^ Impaired in autophagy resulted in the accumulation of damaged mitochondria which further leads to an overproduction of reactive oxygen species (ROS).^35^ ROS attacks DNA and other macromolecules, further impairing HSC function. It has been well-established that ROS increased in aged HSC and leads to the decline of HSC function, moreover, decreasing the concentration of ROS via anti-oxidative agents such as NAC can improve HSC function.^36–38^ In addition to exacerbating DNA damage and subsequently inducing inflammation, the increase of ROS can also directly activate NLRP3 inflammasome, thereby promoting the maturation and release of IL-1β.^39^ Beyond regulating mitochondrial function and ROS production, the autophagy machine itself also has a role in inflammation control.^40^ Previous studies have shown that the conditional knockout of the essential component of autophagy P62 in macrophages leads to the accumulation of damaged mitochondria, activating inflammasome, and cell death.^41^ In another study, researchers have shown that the deletion of ATG16L1 in hematopoietic cells renders mice unable to control LPS-induced inflammation, lacking ATG16L1 in macrophages resulted in overproduction of IL-1b and IL-18 upon LPS stimulation.^41^ However, whether the impaired autophagy exacerbates the inflammatory microenvironment of old bone marrow is still not well understood.

Mitochondrial stress is a well-established driven force of stem cell aging. The dysfunction of mitochondrial metabolic checkpoint renders HSCs escaped from dormancy. In a recent study, Luo at al reported that mitochondrial stress can also trigger inflammation via the NLRP3-Caspase-1 cascade and render HSCs enter pyroptosis. SIRT2, a repressor of the NLRP3 inflammasome, decreased expression in aged HSCs. Forced SIRT2 or inactivated NLRP3 rejuvenates aged HSCs.^[42]^

## 4 FUTURE PERSPECTIVES

### 4.1 Universal aging-related signaling pathway?

The aging inflammatory environment result from multiple inflammatory cytokines upregulation. In principle, one cytokine engages with a specific receptor, can activate multiple different cytoplasmic signaling pathways. Similarly, one signaling pathway could respond to different cytokine receptors. For instance, both IL-1β and TNFα can activate NF-kB,^43^ and the IFN family commonly activates the JAK-STAT signaling pathway.^44^ An intriguing phenomenon is that the deficiency of cytokine receptors generally does not influence the development of mice in physiological conditions (such as *TNFR1/2*^-/-^ mice^11^ and *Il1r*^-/-^ mice^[14]^), however, impairment of intracellular signaling pathways usually results in embryonic lethal (for example *p65*^-/-^ mice^[45]^). It suggests that different cytokines may functionally compensate each other. Counter to cytokines, the transduction of intracellular signaling pathways may be more specific and irreplaceable. In the aging context, whether multiple up-regulated cytokines deteriorate HSC function only through one or a few signaling pathways? If this hypothesis is true, for rejuvenating aged HSCs, the strategy that developing specific inhibitors targeting universal aging-related signaling pathways may be more effective than that eliminating specific inflammatory cytokine in the aged bone marrow.

### 4.2 The initiator of the inflammatory environment?

A long-standing question is what triggers the aging-associated inflammatory environment? Blood cells need to replenish continuously. This process includes the differentiation of HSPCs to generate mature blood cells, and the programmed cell death of senescence blood cells to maintain the homeostasis of the blood system. During aging, the pro-inflammatory cell death pathway may be the dominant manner of blood cell death. In the reproductive system, blocking of necroptosis rejuvenated the aged male.^46^ In another recent study, inactivated Caspase-1, the activator of pyroptosis, rejuvenated aged HSCs.^42^ In this regard, we propose a possible initiator: pro-inflammatory cell death. Beyond the canonical apoptosis pathway, some new forms of pro-inflammatory cell death pathways have been discovered in recent years. Among the pro-inflammatory death pathways, Necroptosis and pyroptosis are the 2 main pathways in which molecular mechanisms were well-established. Necroptosis is mediated by RIPK1-RIPK3-MLKL cascade, which response to the activation of TNFR, TLR, and IFNR, triggering cell death and releasing a large amount of damage associated molecular patterns (DAMPs).^47^ DMAPs derived from blood cells can trigger a secondary inflammatory response and lead to further deterioration of the inflammatory environment. Pyroptosis is mainly mediated by Caspase-1/11-GSDMD cascade, in response to intracellular LPS stimulation, and triggers cell death together with severe releases of IL-1β.^48^ Since genes involved in necroptosis and pyroptosis commonly expressed in blood cells, the inflammatory cell death of blood cells under physiological or pathological conditions may be the initiated causes of the aging-associated inflammatory microenvironment.

### 4.3 Aging-related clonal hematopoiesis

During the aging process, a small fraction of HSCs carrying genetic mutations gain a selective advantage and clonally expand to generate a large number of mature blood cells. This phenomenon is termed clonal hematopoiesis which is a major character of aged hematopoiesis system.^49^ However, the mechanism of how aging promotes clonal hematopoiesis is still largely unknown. Some mutations occur in epigenetic genes (such as DNMT3A, TET2) are considered to be the endogenous drivers that causes clonal blood production.^50^ Whether exogenous factors such as the inflammatory microenvironment promote this process is still unclear. A possible hypothesis is that mutations in specific epigenetic genes render HSCs more resistant to the aging-related inflammation, resulting in the selective advantage of such HSCs during the aging process, which ultimately leads to clonal hematopoiesis. Why epigenetic gene mutations bring the selective advantage to HSCs that render there are resistant to the inflammatory environment eventually leads to clonal hematopoiesis, and, conversely, whether clonal hematopoiesis itself is also the reason of the inflammatory microenvironment in aged bone marrow, these questions need to be further investigated in the future thoroughly.
